# Supplementary material for: Are carbon emissions trading and green financial instruments synergistic? -Comprehensive quantitative research based on content analysis
Source: PLoS One. 2024 Mar 7;19(3):e0298601. doi: 10.1371/journal.pone.0298601 (PMC10919601; doi:10.1371/journal.pone.0298601)
Supplement: S1 Data — (ZIP) [file pone.0298601.s001.zip › Data/地级市Z_F工作报告.zip!/╣π╬≈/└┤▒÷╩╨/└┤╫╘╣1⁄2╓┌║┼í╛┬φ┐╦╩2╛▌═°í┐_2019.docx]

各位代表：

现在，我代表市人民政府，向大会报告工作，请予审议，并请各位政协委员和列席会议的同志提出意见。

一、2018年工作回顾

回顾2018年，困难比预想的多，但发展比预想的好。面对错综复杂的经济形势和艰巨繁重的发展任务，全市上下在自治区党委、政府和市委的坚强领导下，坚持以习近平新时代中国特色社会主义思想为指导，深入学习贯彻党的十九大和十九届二中、三中全会精神，全面落实“三大定位”新使命和“五个扎实”新要求，坚持稳中求进工作总基调，统筹落实“六稳”工作，有效应对和防范化解各种风险，以庆祝改革开放40周年和自治区成立60周年为契机，按照市委“明大势、抓重点、补短板、强弱项、守底线、用干部、善作为”的总体思路，做强“三块长板”，补齐“三块短板”，在压力下砥砺奋进，在困难中开拓前行，有效稳住了经济平稳发展的良好势头。全年主要经济指标先后27次进入自治区“红榜”，县（市、区）100次进入“红榜”。全年地区生产总值同比增长7.2%，高于全区水平，排全区第6位，比上年前进3位，取得自2012年以来最好排位；规模以上工业增加值增长4.7%，其中体现高质量发展导向性指标的工业技改投资首次进入“红榜”；固定资产投资增长18.2%，高于全区7.4个百分点，排第3位，5000万元以上项目投资连续8个月实现30%以上的高速增长，保持全区“红榜”第1位；社会消费品零售总额增长9.6%，高于全区水平，排第4位；进出口总额增长8.7%，排第7位；财政收入50.73亿元，增长5.2%，增幅由负转正，高于上年8.6个百分点。据自治区绩效考评初步结果反馈，我市八大综合性经济指标得分率85.65%，为十年来最高。经过大家“撸起袖子加油干”，我市经济发展已总体走出近年来低谷徘徊的被动局面，部分主要指标已恢复或接近建市以来最好水平，呈现出稳中有进、稳中向好、稳中提质的发展态势，圆满完成了全年目标任务，成绩来之不易。

回顾2018年，压力比预想的大，但应对比预想的稳。面对打赢三大攻坚战的严峻形势和各种风险，我们负重前行，尽锐出战，精准施策，落细落实。积极防范化解政府债务风险，面对在发展中形成的历史债务和密集到来的还债高峰期，我们不回避、不埋怨，沉着应对、主动出击，千方百计、千辛万苦，长短结合、标本兼治，努力争取上级和金融机构、社会各方的大力支持支援和理解谅解，牢牢守住不发生区域性债务风险底线。聚焦脱贫攻坚，突破短板、补齐弱项，扭转了脱贫攻坚战在全区排名靠后的被动局面，金秀县、武宣县有望脱贫出列。集中整治环境污染，狠抓中央、自治区环保督察反馈问题整改，采取全方位、全覆盖、全天候强有力措施，大气污染防治和水环境保护等专项整治工作，获得自治区政府通报表扬和自治区领导充分肯定。事非经过不知难，可谓“看似寻常最奇崛，成如容易却艰辛”。

回顾2018年，负担比预想的重，但惠民比预想的实。面对经济下行压力和财政资金紧缺，我们坚持把有限可用和上级支持的资金毫不犹豫地投向民生和社会事业，顺应人民群众对美好生活的向往，做到不忘初心、勤政为民。开工和建成了一批中小学校幼儿园和城乡基础设施，打通和硬化了一批“断头路”和“水泥路”，组建了一批医联体和医共体，开通了一批村级卫生室便民“村医通”，完成了一批危房改造和棚户区改造，整治了一批城市“五乱”行为，配套完善了一批商贸物流购物便民设施，持续不懈地解决群众反映突出的“出行难、上学难、看病难、购物难、停车难”等烦心事、操心事。全市社会稳定有序，群众安全感持续排名全区前列。人民群众的获得感、幸福感、安全感持续增强。

一年来，我们狠抓2018年《政府工作报告》各项目标任务的落地落实，细化任务清单，按路线图推进，全程跟踪督查，努力践行和兑现向人大代表和人民群众的承诺。据市人大委托第三方对《政府工作报告》的评估结果，565项分解指标的完成率为86.5%，比上年提高8.8个百分点。

一年来，我们主要做了以下工作：

（一）加快新旧动能转换，工业经济实现稳步提升

传统产业转型提档。推进“铝电结合”“热电联产”协同发展，大工业区域电网和东线集中供热管道初步建成并投产运行，银海铝二期实现达产满产，拉动铝、电产业产值分别增长76%、31.3%，较上年提高29.3、42.6个百分点；带动2家铝精深加工企业进驻园区，可增加产值21.95亿元、税收4677万元；热电联产实现向46家企业集中供热，年供热68万吨，实现产值8000万元，带动12家用热大户进驻园区，可增加产值61.03亿元、税收2.99亿元。推进糖企加快重组，东糖、南糖、广糖、湘桂集团等龙头糖业企业形成鼎立之势，预计2018/2019榨季进厂原料蔗900万吨、产糖108万吨、产值60亿元，分别比上榨季增加85万吨、7万吨、2亿元。推进冶炼行业改革重组，中信大锰成功收购汇元锰业，企业保持稳定增长；推进广铁改革重组，挖掘潜力，刮骨疗伤，促进企业在艰难中减亏损、求发展、再重组；中金岭南三期开工建设、推进顺利，来冶、敏诚公司保持稳定生产。全年全市申请直购电53.8亿度，比上年增加7.8亿度，为企业特别是用电大户降低了成本。新兴产业快速成长。推动碳酸钙产业发展，建成规上企业46家，产值达到52亿元，增长13%。引进新东运、汇宾科技、日昌升等绿色环保型碳酸钙龙头企业8家，投资额达117.3亿元，建成投产后可新增产值114.06亿元、税收13.23亿元，碳酸钙产业有望迅速成为我市新兴的支柱产业。加快培植发展建筑新材料、节能环保、电子信息、生物制药和瑶医瑶药、汽车摩托车配件等新兴产业，中沛、铭磊、汇萃本草、大联统摩托车等一批龙头企业相继签约进驻和开工竣工。全市共引进新兴产业企业9家，投资总额69亿元，可新增产值104亿元、税收4.13亿元，为我市工业“建树成林”和新旧动能转换注入了新动力、新活力。服务企业落细落小。抓好规上企业培育工作，全年新增规上企业27家，较上年增加5家。新签约工业项目64个，新开工45个，实现竣工15个。投入8.09亿元完善园区基础设施建设，工业投资实现增长24%，有效摆脱了近年来负增长的局面。对全市规上企业和培规企业进行全程跟踪走访服务，跟踪督查和协调解决问题268个，为企业降低用电、用气、用热等各类成本18.4亿元，促进规上企业利润由去年亏损1.95亿元转为盈利5.28亿元。创新能力接续增强。通过国家高企备案认定企业10家、高新技术企业26家，现有孵化器、众创空间7家，新增在孵企业32家，增长43.2%。有效发明专利拥有量314件，增长10.9%。实现科技成果转化15项。获批创建国家农业科技园区、自治区瑶医瑶药创新小镇。象州县荣获“广西科学发展进步县”。

（二）围绕乡村抓振兴，现代农业不断提质增效

农业基础设施日臻完善。水利固定资产投资26.6亿元，农田灌溉面积保持在140万亩左右。全面完成桂中治旱工程征拆任务，专项迁改顺利推进，部分工程发挥效益，一、二期工程分别完成总投资的97.5%、74.2%，基本扭转了进度偏慢的被动局面。加快大藤峡移民安置工程建设，武宣县城防护工程及移民安置点建设有序推进，全年完成投资10.22亿元，累计完成投资41.35亿元，占总投资的61%。农业产业稳步增长。荣获全国首批认定的全国首个地级市国家现代农业产业园，引进正大、新希望、光大、鲁美等一批知名企业进驻产业园。农林牧渔业增加值增长5.8%，较上年提高1.2个百分点，排全区第3位。粮食产量70.56万吨，继续保持稳定。新建“双高”基地55万亩，总规模达95万亩。糖料蔗、水果、蔬菜、肉类、水产品、木材产量分别增长9.1%、31.7%、5.6%、3.4%、6.2%、9.9%。农业发展质量不断提升。新创建各级示范区357个，其中自治区级7个、县级21个、乡级69个、村级260个。新增农民专业合作社和家庭农场905家，新获评广西示范合作社、示范家庭农场19家。24个农产品和8个富硒产品分别通过“三品一标”认证和富硒认证，农产品质量安全例行监测合格率98%以上，水产动物产品安全例行监测合格率100%。农业机械总动力达220.4万千瓦，农业农作物耕种收综合机械化水平达60.5%。荣获全国“平安农机”示范市，忻城县荣获全国“平安农机”示范县，兴宾区、象州县纳入全国“中国好粮油”示范县和重点县。农业农村环境持续改善。圆满完成“美丽来宾•宜居乡村”活动，建成市级“宜居乡村”综合示范村41个，完成改厕、改厨、改圈12万多户。

（三）发挥特色资源优势，服务业发展日趋旺盛

商贸流通日趋兴旺。百悦商业、红星美凯龙等商业综合体投入使用，来宾特色产品超市、特色街、汽贸园等竣工开业，城区商圈服务功能进一步完善；出台扶持培育限上企业政策，培育限上企业20家。实施电子商务、电商精准扶贫“三年行动计划”，大力实施“电商来宾”工程，兴宾区电商产业园实现开园，忻城县、金秀县商务部电子商务进农村示范县建设扎实推进，全市村级以上电商服务点达到546个。国家电信普遍服务试点建设取得突破性进展，新建通讯基站1387个，全市网络覆盖率达94%，电信业务总量实现翻番，增长2.5倍。邮政业务总量增长28.7%，较上年提高1.8个百分点。完成商品房销售面积179.6万平方米，增长12.8%，较上年提高8.4个百分点。金融机构存款余额724.2亿元、贷款余额534.29亿元，存贷比73.8%。全域旅游升温提质。打造“壮歌瑶韵•畅游来宾”等假日节庆旅游品牌，新增国家级3A景区3个，新增星级乡村旅游区15家。创造民宿旅游“金秀模式”，全县营业民宿达49家。金秀县创建国家全域旅游示范区通过自治区初审，荣获广西首个“中国天然氧吧”称号；忻城县获列为广西特色旅游名县创建县，象州、武宣、忻城、合山入列自治区级全域旅游示范区创建单位。全年共接待国内外游客2832. 5万人次，增长25.2%；旅游总消费243亿元，增长34.3%，较上年提高3.3个百分点。交通路网持续织密。柳南高速公路改扩建工程（来宾段）建成通车，柳州经合山至南宁、贺州至巴马（蒙山至象州段）一期工程高速公路实质性动工。象州石龙至来宾、百丈至大樟等一批路网项目进展顺利，完成187个农村公路项目建设，建制村通畅率、通客车比例均实现100%。完成交通固定资产投资34.5亿元，公路客货运周转量增长9%，水路客货运周转量增长27.5%；港口货物吞吐量突破2000万吨大关，增长81.7%，较上年提高73.2个百分点。全市服务业增加值增长9.9%，高于全区水平，排全区第8位，对经济增长贡献率超过五成。

（四）突破重点攻克难点，三大攻坚战初战告捷

防范化解重大风险攻坚战管控稳妥。深入开展互联网金融风险和非法集资风险专项排查活动，对5家企业进行“预警”，2家企业进行“立案调查”，金融风险防范能力有效提升。将土地开垦和“旱改水”项目建设作为可持续发展的重要举措，共立项项目58个，为推动乡村振兴、筹集发展资金、摆脱发展困境探索了新路径。规范政府举债融资行为，积极争取上级财政转移支付、专项债、收益债等支持，加快存量土地、烂尾楼和闲置资产处置，压缩部门财政预算，控制政府投资规模，建立防范债务风险资金池、完善应急处置机制，向上级争取政府债券资金30.61亿元，共化解全口径债务107.67亿元。精准脱贫攻坚战扭转困局。聚焦脱贫攻坚政策、责任、工作“三落实”，扎实打好“五场硬仗”和做好“七大重点”工作，预计减少农村贫困人口8.3万人、96个贫困村出列，金秀县、武宣县有望脱贫摘帽。完成基础设施建设项目473个，投入产业扶持资金2.73亿元，特色产业覆盖率达95.2%，受益贫困户7.7万户。2017、2018年共26个易地扶贫搬迁集中安置项目全部完工，搬迁入住30612人，综合进度排全区第一；搬迁户转移就业1.06万人，累计投入后续扶持资金2.57亿元。已脱贫和计划脱贫摘帽的189个贫困村集体经济收入均达到3万元以上。全市3.9万名帮扶干部与7.86万户贫困户进行全覆盖结对帮扶。金融、健康、教育、电商扶贫等工作扎实推进，粤桂扶贫协作成效显著。忻城县“易地搬迁拔穷根”项目荣获全国民生示范工程奖。污染防治攻坚战守牢底线。狠抓中央环保督察“回头看”交办的122件信访案件、自治区环保督察组交办的146件信访案件的查处整改，督办案件均在规定时限内办结。开展大气污染“百日攻坚”专项行动，强化烟花爆竹燃放、秸秆焚烧等管控，全年空气质量优良天数比率为83.8%，比上年提高2.15个百分点；PM2.5平均浓度下降16.66%，全区降幅最大。严格落实河长制、湖长制，地表水各断面水质、在用集中式饮用水水质达标率均为100%。完善危险固体废物监管联动机制，查处了2起跨区域固废转移重大案件。推进重金属污染防治与修复，加强污水垃圾设施建设，清理农村垃圾20万吨，清洁水源1.29万处，清捡田园面积22.9万亩。成功创建全区唯一一座国家级绿色矿山。环保工作获自治区领导200万元专项工作经费支持。一年来，我们切身感受到奋战在攻坚一线广大干部群众的艰辛，他们真抓实干、埋头苦干，“5+2”“白+黑”连续作战，我们为他们不畏艰难、不畏劳苦的敬业精神点赞！

（五）做大有效投资增量，发展后劲趋实向强

营商环境向优向好。打造“来宾效能快车”， 出台“1+5”优化营商环境系列文件，推行“242530”改革，企业开办、不动产抵押登记等办理速度较全区再提速20%~50%，较法定办结时限提速80%~88%，是全区改革力度最大、提速幅度最大的设区市之一；积极推进“一事通办”和“网上办事”，全市“最多跑一次”事项比例达88%，市本级90%以上事项可网上办理。推进商事制度改革，推行“注册资本认缴”“先照后证”“多证合一”，市场主体增加1.87万户，增长9.6%。建立政府性融资担保体系，为小微企业提供贷款担保2.37亿元；开展银企对接会8次，促成银企贷款30亿元。精准招商再结硕果。充分发挥“一网两管”优势，在资源换产业上下功夫，持续开展“一把手”招商，采取补链招商、以商招商、驻点招商等方式，成功引进一批国内知名企业，三江口港产城新区形成由龙头企业带动实施连片开发的招商开发新模式。全市新签约正式协议95个，总投资773.57亿元，实现到位资金502.32亿元，较上年增长15.1%，其中亿元以上产业项目72个。项目建设保持领先。编制三年滚动计划项目450个总投资4175亿元，在全区最先将165个总投资932.8亿元项目纳入第一批新西部大开发基础设施补短板项目盘子。召开投资和项目专题协调会12次，协调解决问题160多个。争取到中央预算内项目补助资金12.2亿元，新增建设用地指标1万多亩，获得国务院用地指标奖励5000亩。新东运、中沛二期等146个项目实现新开工；双飞汽配、象州航桂发电等75个项目竣工投产；中沛电子产业园、电厂生活区片区旧城改造等一批在建重大项目加快建设。民营经济发展加快，民间投资增长27.2%，提高19.8个百分点。

（六）狠抓关键领域改革，发展活力持续释放

供给侧结构性改革稳步推进。有效实施去产能，排查并逐步清除 “僵尸企业”6家，关闭煤矿4对井，退出煤矿产能66万吨。防范风险去杠杆，实现中小微企业贷款余额234亿元，增长13.3%。减税降费降成本，组织119家企业参加电力市场化交易，降低用电成本5亿元。聚焦民生补短板，完成基础设施建设投资152.96亿元。重点专项改革不断深化。推进农村土地“三权分置”改革，完成确权承包地358.3万亩，实现承包经营权证可颁证率97.4%，排全区前列；合山被确定为全国农村土地承包经营权确权登记颁证图纸档案整理与保存项目试点单位；象州被列为全国农村土地承包经营权抵押贷款试点县。稳步推进国有农林牧场改革，将全市原12家县属国有林场整合为8家。持续深化国企国资改革，提升了国有企业防范金融风险能力和经济竞争力。推进医药卫生体制改革，不断完善医疗联合体建设机制，提升了市、县两级公立医院服务能力，县域内就医率达90%以上。

（七）注重城乡一体化建设，县域经济协同发展

城市承载能力增强。市主城区建成区面积扩大到50.66平方公里，常住人口城镇化率44.55%，比上年提高0.79个百分点。持续推进城北新区完善提升配套服务功能建设，建设82个项目总投资39.3亿元，建成一批道路交通、中小学校、商贸娱乐等设施。推进国家新型城镇化综合改革试点等工作，新增城镇常住人口2.43万人。人居生活环境趋优。完成第二批立体绿化工程，城区绿化覆盖率达32.8%；新增海绵城市建设面积2.67平方公里，完成地下综合管廊建设7.44公里；盘活存量建设用地19603亩，处置闲置土地1095亩，6个房地产停建项目恢复施工。碧桂园、云星等高品质房地产进驻我市。石龙镇、思练镇培育自治区级特色小镇和桐木镇培育自治区创新小镇试点有序推进。迁江镇获列入全区第一批经济发达镇行政管理体制改革范围。县域经济发展加快。兴宾区加快推进现代农业、碳酸钙、制糖等重点产业发展，生产总值增长9.4%；金秀县大力发展生物医药、旅游等特色产业，生产总值增长10.8%；合山市着力推动“热电一体”、碳酸钙等产业发展，生产总值增长9.5%；来华管理区和河南工业园区着重推进高新技术产业、铝精深加工、节能环保等产业发展，河南工业园区规上工业增加值增长21.4%。

（八）全力强保障惠民生，群众获得感幸福感安全感更加充实

教育事业加快发展。新（改、扩）建乡镇公办幼儿园20所，来宾市实验高中迁建、校外实践基地、铁北小学等一批中小学校建成办学；来宾一中迁建、盘古小学、幼教集团二幼三幼等一批项目开工建设。义务教育辍学率0.2%，控制在1.5%以内。高考质量稳步提升，一本生源万人上线率由上年全区第12位提高到第8位。实施中等职业学校布局调整和专业结构优化，来宾职业教育中心学校通过国家改革发展示范校验收，被评为广西首批四星级学校。广西科技师范学院在校生突破1.5万人。文体事业蓬勃发展。新建村级公共服务中心66个，新增区级文物保护9处。市县公共图书馆实现国颁等级全覆盖，搭建了“来宾文化云”数字平台。举办群众文化活动2100多场，农村免费播放电影8700多场。公共文化服务体系建设入选纪念改革开放40周年大型政论专题片《必由之路》。成功承办广西“壮族三月三·民族体育炫”暨首届广西体育庙会、“奔跑吧·广西”生态马拉松系列赛（象州站）。我市运动员在各项赛事中取得自治区级以上的金牌23枚、银牌24枚、铜牌52枚的好成绩，被评为第一批广西全民健身和全民健康深度融合示范市。卫生保健特色鲜明。荣获全国无偿献血先进市，金秀县、象州县获评全国基层中医药工作先进单位。市人民医院、中医院分别与区级医院构建医联体，6个县（市、区）与58家乡（镇）卫生院组成县域医共体。落实建档立卡贫困人口县域住院就医“先诊疗、后付费”便民政策，基层医疗信息管理系统上线率和应用率均达100%。全面两孩政策有序实施，人口自然增长率7.79‰。稳步推进妇幼健康工作，免疫规划等公共卫生服务体系不断完善。社会保障殷实有效。城镇新增就业1.51万人，城镇登记失业率控制为2.95%，低于控制目标1.15个百分点。实现城乡低保、特困人员按标施保，应保尽保。开工建设新的烈士陵园。筹措3.34亿元资金，实施铁道口扩建、中南路延长线等10件为民办实事项目。完成棚户区改造开工4226套，基本建成6781套，农村危房改造8307户。居民消费价格总水平控制在3%以内。城镇居民人均可支配收入增长6%，农村居民人均可支配收入增长10.1%。社会治理管控有序。扎实推进扫黑除恶专项斗争、禁毒人民战争等专项行动，扫黑除恶成效排全区第5名，全市刑事、治安案件分别同比下降0.94%、5.3%，市区“两抢”警情下降52.3%，零发案天数324天，群众安全感继续保持全区前列，排第3名。武宣县获评第四批“全国法治县（市、区）创建活动先进单位”。全市信访批次、人次分别下降9.2%、13%，新发“三大纠纷”案件调结率93.6%。开展社区矫正安全稳定“百日”专项行动，特殊人群教育管理扎实有效。安全生产形势稳定，达到全年事故防控目标。全年无重大食品药品安全事故，形势总体稳定向好。圆满完成自治区成立60周年各项筹备工作和庆祝活动，得到中央代表团的充分肯定。

一年来，我们切实加强政府自身建设，认真落实意识形态工作责任制，坚定不移地推动政府系统全面从严治党向纵深发展，严格执行中央八项规定精神，实行政府党组成员分管部门警示教育负责制，实现政府各工作部门、各直属机构廉政警示教育全覆盖；加强自治区党委巡视组反馈意见整改，涉及市政府系统25项整改任务已基本完成；坚持依法行政，扎实推进法治政府建设；认真贯彻市委决策部署，自觉接受人大的依法监督、政协的民主监督和社会的舆论监督，认真落实市人大常委会对市人民政府各专项报告的审议意见，办理人大代表意见建议79件、政协委员提案117件，办复率达100%。

审计监督、依法统计、政务督查、政务公开和应急管理等工作扎实推进。国防动员、人民防空、民兵预备役、优抚安置、征兵等工作继续加强，双拥模范城创建成果得到深化巩固，军民融合进一步发展。机关事务、民族宗教、外事、科协、侨联、供销、气象、地震、工会、共青团、妇联、工商联、档案、地方志、残联、红十字会等工作各有特色亮点，都取得新的成绩。

各位代表，单丝不成线，独木难成林。过去一年成绩的取得，是市委统揽全局、坚强领导的结果；是市人大、市政协有力监督、大力支持的结果；是各位人大代表、政协委员理解信任、建言献策的结果；是全市广大干部群众和社会各界同心协力、砥砺奋进的结果。在此，我代表市人民政府，向全市各族人民，向人大代表和政协委员，向各民主党派、工商联、无党派人士、人民团体和社会各界人士，向驻市的中直、区直单位和人民解放军、武警官兵，向所有关心和支持来宾发展的港澳台同胞、海外侨胞和企业家、外来投资者、国内外友好协作市县和在外打拼创业的来宾同乡，表示衷心感谢并致以崇高敬意！

各位代表，船到中流浪更急，人到半山路更陡。我们清醒地认识到，经济社会发展仍存在不少问题和短板。一是稳增长高质量发展的结构性、深层次矛盾尚未得到根本上解决，存在较多“难点”。新旧动能转换不足，产业发展步履艰难，糖、铝、冶炼等传统产业转型步伐偏慢，高耗能行业占比仍高达60%以上；碳酸钙、茧丝绸、印染服装、汽配等新兴产业体量较小，对工业增长贡献率不到1个百分点；规上工业增加值、全口径税收收入等稳增长基础性指标、高质量发展导向性指标不够突出，“红黑榜”排位仍是“不红不黑”居多；工业企业因生态环保、经营管理等7个方面问题导致停减产面居高不下，产值负增长面达13.8%，其中负增长超过20%企业面达19.2%，排全区靠前。二是三大攻坚战基础不牢、压力巨大，存在较多“痛点”。财税增长乏力，财力薄弱的状态仍没有改变；历史债务包袱沉重，存在化解压力大、资金筹措缺口大、债务率下降难度大等严峻问题，短期内走出债务风险困难重重；脱贫攻坚基础薄弱，普遍存在产业支撑不足、易地搬迁后续扶持滞后、村集体经济发展后劲乏力等问题，目前尚未脱贫的1个国定贫困县、80个贫困村、8.4万贫困人口大多属贫中之贫、坚中之坚，如期实现脱贫任务十分艰巨；污染防治形势依然严峻，来宾冶炼厂厂区、周边及鸡公山渣场的环境问题去年被中央环保督察组定性为“敷衍应对，纸上整改”，整治任务繁重。“小散乱污”企业整治难度大，大气污染有关指标在全区排名倒数，城乡结合部生态环境问题占比高达80%，治理难度很大。三是营商环境优化不够，存在较多“堵点”。群众办事仍然遭遇手续繁琐、找不着门、跑腿多、等候时间长等问题；企业生产经营仍碰到审批效率低、要素成本高、费用负担重、融资难融资贵等问题。四是落实全面从严治党要求不够实，存在较多“风险点”。工程项目招投标、政府采购、产权转让等关键环节和国有企业领域腐败的风险点仍然存在；群众身边的腐败、扶贫领域腐败和作风问题时有发生；少数干部仍存在不作为、慢作为、不敢为、不会为等问题。对此，我们必须勇于直面问题，不遮掩、不回避、不懈怠，只要我们敢闯敢干、埋头苦干，来宾就没有迈不过的坎、闯不过的关！

二、2019年工作安排

今年是新中国成立70周年，是全面建成小康社会、实现第一个百年奋斗目标的关键之年，也是广西全面贯彻落实习近平总书记重要题词精神的第一年。做好今年的工作，我们肩上的担子非常繁重，但我们的使命无比光荣。今年政府工作的总体要求是：以习近平新时代中国特色社会主义思想为指导，全面贯彻党的十九大和十九届二中、三中全会精神以及中央、全区、全市经济工作会议精神，按照建设壮美广西、共圆复兴梦想的总目标总要求，结合来宾实际，持续深入落实“三大定位”新使命和“五个扎实”新要求，统筹推进“五位一体”总体布局，协调推进“四个全面”战略布局，坚持稳中求进工作总基调，坚持新发展理念，坚持推动高质量发展，坚持以供给侧结构性改革为主线，坚持深化改革优化营商环境，全面贯彻落实市委四届六次全会精神，按照市委“三提升、三攻坚、一优化”的工作思路，统筹推进稳增长、促改革、调结构、惠民生、防风险工作，进一步稳就业、稳金融、稳外贸、稳外资、稳投资、稳预期，保持经济持续健康发展和社会大局稳定，为全面建成小康社会收官打下决定性基础，以优异成绩庆祝新中国成立70周年。

今年经济社会发展的主要预期目标是：实现地区生产总值增长7%~7.5%，财政收入增长3%力争增长5%，规模以上工业增加值增长7%左右，固定资产投资增长10%，社会消费品零售总额增长10%，外贸进出口总额增长8%，节能减排降碳控制在自治区下达目标内，城镇居民人均可支配收入增长6%，农村居民人均可支配收入增长9%，城镇登记失业率控制在4.1%以内，居民消费价格总水平涨幅控制在3%以内，实现忻城县脱贫摘帽，全市农村贫困人口减少5万人以上。

当前我市面临的形势更加复杂，机遇与挑战并存。上述主要目标，是全面贯彻落实市委决策部署，从实现我市“十三五”规划目标、全面建成小康社会，与自治区经济社会发展预期目标相衔接及我市经济发展各项支撑条件等因素综合考虑，绝大部分指标与全区水平持平或略高于全区水平。需要说明的是，财政收入保底增长3%，力争增长5%，确保与去年增长和今年全区增幅相符。总的来说，这些预期目标既坚持了稳中求进工作总基调和实事求是的原则，兼顾了稳妥性和积极性，也体现了推进经济高质量发展要求，体现了“幸福是奋斗出来”的实干精神和“心容天下，敢为人先”的来宾精神，体现了“担当为要、实干为本、发展为重、奋斗为荣”的为政理念，体现了全市人民对美好生活的向往和改变我市落后面貌的期盼和信心。

为政之要，贵在落实。为实现上述目标，我们要紧紧围绕市委“三提升、三攻坚、一优化”工作思路，把“规划图”变为“施工图”，把“时间表”变为“计程表”，锁定目标，倒排工期，撸起袖子，干出样子，决不辜负全市人民的信任和期望！

我们着重抓好以下八个方面的工作：

（一）聚焦做大做强工业，舞起实体经济龙头

没有实体就没有希望，没有产业就没有未来。要应对挑战、破浪前行，实现凤凰涅槃、行稳致远，根本的出路就是要致力于做大实体经济、做强工业产业、做实财税蛋糕。要做大实体经济，关键是要做强工业产业。我们要全面贯彻落实“实体经济发展年”，围绕强龙头、补链条、聚集群，坚定不移培植“工业树”、打造“产业林”，切实落实自治区“1+14”和我市“1+5”营商政策措施、碳酸钙产业发展措施以及工业发展“双十条”和林产品加工业发展若干措施，推动工业高质量发展，实现规上工业总产值增长12%；培育规上企业30家，其中补链条的企业占50%以上。

推进传统产业“二次创业”。通过技术创新和强龙头、补链条，使传统产业由产业链低端末端逐步走向中高端，真正实现转型升级。做实做强铝业“二次创业”，完善区域电网建设，以银海铝业为龙头，重点发展建筑和工业用铝型材、铝板带箔、车用和消费品铸造零部件等下游产业，加快推进广投集团铝合金扁锭、海螺建工建材等补链项目，全力打造铝精深加工基地，实现铝产业产值增长15%以上。做实做强冶炼业“二次创业”，重振冶炼业发展，以广铁、来冶、敏诚矿业等为龙头，完成广铁公司的债转股和战略重组工作，重点支持汇元锰业技改等冶炼企业延链项目建设，实现铝业以外的冶炼业产值增长10%以上。做实做强糖业“二次创业”，继续优化重组中小制糖企业，以东糖、南糖、广糖、湘桂集团为龙头，全力推进糖业全产业链开发；加快来冰功能糖、福斯派环保餐具等项目建设，力争年内竣工投产。实现制糖业增加值增长3%，综合利用业产值占糖业产值的25%以上。

培育壮大新兴产业。充分挖掘资源优势，从优势资源中催生壮大新兴产业。做大做强绿色碳酸钙和建筑新材料产业，以汇宾钙、福美新材料为龙头，推动形成碳酸钙和新材料生产、加工、研发、应用全产业链生产体系，加快推进新东运、华纳新材料等项目建设，实现规上碳酸钙产业产值增长10%；加快推进中钻达新能源新材料、装配式建筑等产业园项目建设。做强做优节能生态环保产业，深化国家环境服务业试点工作，推进热电联产循环经济生态产业园示范基地建设，开工建设三江口节能环保生态产业园，推进雅居乐印染丝绸、北京驰普智能家居、飞南铜资源再生利用等项目建设。做实做强电子信息和先进制造业，重点推进中沛汽车电子、德福特二期、信荣达电子等项目建设，加快推进凤凰汽配产业园、湘电风电制造基地等先进制造业建设，实现电子信息和先进制造业产值增长15%。做精做实健康养生和生物制药产业，推进铭磊医药、维威药业、天顺祥药业、汇萃本草瑶药健康产业等生物制药产业发展，实现生物制药业产值增长15%。

支持民营经济发展壮大。认真落实自治区民营经济“55条”，着力扶持民营企业发展壮大。树立“企业需要时，政府无时不在；企业不需要时，政府无声无息”理念，建立市县领导联系企业机制，全覆盖服务好现有民营企业，定期协调解决民营企业诉求，把服务现有民营企业作为优化营商环境的重中之重。开展民营经济示范县、乡和示范园区创建活动。实施民营企业强龙头培育计划，兴宾、象州、武宣、市工业园区分别选定不少于5家重点龙头型企业、10家成长型骨干企业、10家中小企业，其他县（市）也分别选定一批企业进行培育。营造公平竞争环境，对各类企业一视同仁，禁止在工程招投标、政府采购、国有土地和矿权出让、国有产权交易等过程中设置限制或者排斥民营企业的不合理条件。支持民营资本组建或者参股相关投资资金，参与PPP项目建设，参与乡村振兴建设，实现民间投资增长10%以上。积极兑现优惠政策承诺，全力维护民营企业合法权益。切实维护企业家人身财产安全。民营企业家是我们自己人，我们一定像对待自己的亲人一样关心民营企业，一定像对待自己的兄弟一样帮扶民营企业家，让来自五湖四海的天下“来宾”真正成为“来者上宾”！

实施科技创新驱动。把科技创新摆在工业高质量发展的重要位置，围绕打造“六张创新名片”，申报科技计划项目40项以上，新增国家高新技术企业5家以上，实现科技成果转化11个以上。推进双创平台建设，提升现有5家市级以上科技企业孵化器、众创空间服务能力，在孵企业60家以上，40%以上的在孵企业配有创业导师；推进广西非金属矿物工程技术研究中心建设。加大工业技改力度，提高“千企技改”数量，投资增长20%以上。

（二）聚焦推进乡村振兴，掀起农业农村现代化建设热潮

乡村振兴是民之所望、政之所向，必须稳住“三农”这个基本盘，扎实推进农业农村现代化，实现农林牧渔业增加值增长5%。

推动农业全面升级。以我市获得认定国家现代农业产业园为新起点，全力推进现代特色农业全产业链发展。做大做优现代农业特色产业和农林产品加工业，以国家现代农业产业园为龙头带动，推进正大生猪全产业链、光大牧业羊全产业链、鲁美构树饲料、邦禾蔗果全产业链等重大项目建设，加强凤凰、良江农产品加工园等集聚区和林产品加工园建设，提高种养经济效益；创建各级示范区310个以上，其中自治区级3个、县级5个、乡级50个、村级252个。落实好粮食安全行政首长责任制，推进高标准农田建设50万亩，实现粮食产量70万吨以上。推进水果、蔬菜、水产草食畜牧等种植养殖业发展，提升规模化、标准化、产业化、生态化程度，实现水果、蔬菜、肉类、水产品产量分别增长20%、3%、2.5%、4%。新增糖料蔗“双高”基地10万亩，全面完成105万亩建设任务，实现“双高”基地耕种收综合机械化水平75%、机收率30%以上，2019/2020榨季进厂原料蔗增长6%以上，农作物耕种收综合机械化率62.5%以上。落实油茶“双千”计划，推进油茶产业发展。加强农产品质量安全监管和品牌建设，农产品质量监测合格率97%以上，全力打造国家现代农业产业园农产品区域公用品牌，推进沃柑、红茶等品牌建设，争取30个农产品通过“三品一标”认证。

推动农村全面进步。深化农村土地制度和产权制度改革，建设完善农村产权交易市场，探索开展土地经营权抵押贷款试点工作，发展壮大村集体经济，全市村集体经济收入3万元、5万元以上的行政村分别达491、239个。激活农村发展新动能，全面推进农村人居环境整治三年行动，启动乡村风貌提升三年行动，抓好金秀试点县工作，大力推进“美丽来宾·幸福乡村”建设，开展“环境秀美”“生活甜美”“乡村和美”三个专项活动，打造40个市级“幸福乡村”综合示范村。按上级时间要求，全面完成“大棚房”清理整治。严格落实“林长制”，实施植树造林20万亩，森林覆盖率达52.73%。

推动农民全面发展。加大本土人才培养力度，积极推进新型职业农民培育和青年农场主培养工作，完成新型职业农民培育1000人以上，深化开展“万才返乡•共建小康”活动，制定激励优惠政策，鼓励外出能人返乡创业，鼓励大学生村官扎根基层，形成各方参与产业开发、乡村建设的良好氛围。重视新型经营主体发展质量，打造自治区示范合作社、示范家庭农场20家以上。

（三）聚焦创特色树品牌，蓄势加快现代服务业发展

正视我市实际，坚持“不求最大，但求最特”的理念，以“特”领先，以“特”取胜，借助一二产业的“势”发展现代服务业，实现服务业增加值增长9.5%。

提升集聚发展能极。着力创建滨港现代物流、兴宾区电子商务等自治区级服务业集聚区，继续提升金秀瑶都生态养生服务基地、高新科技园现代服务聚集区发展，引导和促进现代服务业向集聚区发展。围绕服务产业园区和碳酸钙产业发展需要，加快兴宾、象州、武宣港口码头建设步伐，大力发展现代物流产业。年内公路客货运周转量、水路客货运周转量、港口货物吞吐量要分别增长8%、18%、10%以上。

促进消费提档升级。培育和打造新旧城区商贸圈、餐饮特色街等消费热点，支持来宾特色产品超市发展壮大，打响“天下有来宾，来宾有特产”特产品牌。抓好桂中建材、来宾汽贸园等的招商引资和配套服务，进一步挖掘市场消费潜力。大力培育“小上限”企业，全年新增限额以上企业15家以上。实现批发、零售业销售额分别增长10%、15%，住宿、餐饮业营业额分别增长10%、10%。加大金融服务力度，存贷款增速均达12%以上。深入实施电子商务三年行动计划，加快农村电子商务服务体系建设，实现电子商务进农村覆盖率达40%以上。推动5G发展，电信业务总量增长190%以上。推动全域旅游发展。借鉴“共享农庄”理念，在金秀探索“共享村落”“共享民宿”创建试点。扎实推进象州、武宣、忻城、合山创建自治区级全域旅游示范区和广西特色旅游名县工作，完成武宣百崖大峡谷、金秀古沙沟景区创国家4A级景区评定，新增乡村旅游区9家以上，力争成功创建金秀县国家全域旅游示范区。全年接待游客达3300万人次，增长16%以上，旅游总消费315亿元，增长30%以上。

加快数字经济建设。积极创建数字经济产业园，启动数字经济示范城建设，加快编制《广西数字化经济发展示范区（来宾）五年规划》，推进数字经济社会发展三年行动计划，推动互联网、大数据、人工智能与实体经济深度融合，加快冶金、能源、铝及精深加工、制糖及综合利用等传统优势产业数字化改造升级。实施数字来宾“广电云”村村通户户用工程，实现“广电云”村村通。全面落实宽带网络提速降费政策，全市宽带普及率达到70%。加快数字产业培育，抓住5G商用机遇，发展5G装备制造业。

（四）聚焦补短板强弱项，蓄力提升基础设施水平

抓住国家对中西部补短板扩投资的契机，主动融入全局谋项目，着力补齐园区和交通设施短板，实施项目170个以上，投资60亿元以上。

着力补齐工业园区配套设施短板。积极引入社会资本和龙头企业带动园区建设开发。重点推进河南园新科路、迁江园人才公寓小区建设；推进高新园区路网、“三产”安置小区一期项目建设；推进凤凰工业园区跨湘桂铁路高架桥、园区至凤凰污水处理厂排污项目建设；加快三江口港产城新区建设，开工建设正龙红水河大桥、莆田红水河大桥、黄茆黔江大桥等石龙片区、高安片区基础设施建设；完善兴宾高安新材料、兴宾区碳酸钙、合忻碳酸钙和各县（市、区）林产品深加工等园区的水、电、路、污配套设施，不断提升园区承载力和发展效益；抓好大工业区域电网广铁线建设，扩大供电范围；加快推进金秀桐木、忻城红渡、武宣黔西等工业园区污水处理厂建设。

着力补齐农业基础设施短板。全面落实国家现代农业产业园发展规划，充分利用好国家的资金和政策支持，进一步完善全国蔗糖全产业链升级引领区、全国蔗果畜加生态大循环示范区、广西农业结构调整与产业融合升级样板区设施建设，加快完善凤凰和良江农产品加工园区等水电路及通信设施建设。深入实施现代特色农业示范区建设增点扩面提质升级行动，进一步完善自治区、县、乡级现代特色农业示范区设施建设，提高农业产出率。加快推进桂中治旱工程建设，6月底前实现一期工程总干渠贯通，完成溯河、环山支渠建设，分段发挥忻城、合山片区4.8万亩以上灌溉效益；二期工程完成投资3.2亿元；抓好大藤峡库区移民安置，完成投资5亿元；加快推进下六甲灌区前期工作，继续推进病险水库除险加固工程建设，实现水利投资增长10%。

着力补齐交通路网建设短板。推进高速公路建设，加快推进柳州经合山至南宁高速公路（来宾段）、贺州至巴马高速公路（蒙山至象州段）一期工程建设，新开工贺州至巴马高速公路蒙山至象州段二期工程、象州至来宾段、来宾至都安段，着力构建“两横四纵一环”高速公路交通网络。完善城乡路网建设，加快象州石龙至来宾、蒙山至金秀等路网项目建设，推进“乡乡通二级公路”进程。推进国家现代农业产业园“一横三纵”公路干线道路拓展升级。加强金秀全域旅游基础设施建设。完善港口物流设施，推进来宾港及武宣港区大仁、龙从作业区工程建设。

（五）聚焦风险防控打硬仗，扛起打好三大攻坚战政治责任

打好三大攻坚战，还有不少硬骨头要啃、重点战役要打，每一战都不是敲锣打鼓、轻轻松松就能实现的，我们必须要有敢打硬仗的勇气和敢闯难关的韧劲。

全力化解重大风险。坚持底线思维，增强忧患意识，提高防控能力，着力防范化解重大风险。特别要通过“提升融资效率、压缩政府性投资、加大资金筹措、建立防范债务风险资金池”等举措，着力防范和化解债务风险，切实做到“坚定、可控、有序、适度”。树牢过“紧日子”思想，压减一般性支出6%以上。加大土地运营力度，有效整合国有林场、矿权等资源，加快处置闲置资产，大力实施土地开垦和“旱改水”项目，开辟推进乡村振兴、筹集发展资金、摆脱发展困境新路径。积极争取新增政府债券，切实提高化债能力。加强薄弱环节监管制度建设，加大非法金融活动排查整治工作力度，坚决打击违法违规金融活动，牢牢守住不发生系统性区域性金融风险的底线。

全力冲刺脱贫攻坚。围绕“两不愁、三保障”总体目标，举全市之力，集全市之智，聚焦深度贫困地区和特殊贫困人口如期脱贫，进一步完善脱贫攻坚责任体系，设立18个行业扶贫指挥部，建立市级打赢忻城县脱贫摘帽攻坚战包村责任制，统筹社会资源强力攻坚，如期完成年内减贫5万人以上，67个贫困村、忻城县脱贫摘帽。实施脱贫攻坚“春季攻势”十大行动，全力打好“七场硬仗”，突出推进“十一大重点”工作，深入实施特色产业提升工程，构建完善县级“5+2”、贫困村“3+1”特色产业体系，实现产业扶贫对有发展能力的贫困户全覆盖。加快推进易地扶贫搬迁项目建设，实现搬迁1059人，强化后续产业发展和就业创业扶持，确保搬迁一户、脱贫一户。大力发展壮大村级集体经济，确保年内预脱贫摘帽的贫困村集体经济收入达到4万元以上。统筹推进贫困村屯道路、安全饮水、电力改造、宽带网络和村级公共服务等基础设施建设。深化粤桂扶贫协作，健全茂名—来宾扶贫协作精准对接机制，推动各方面协作取得更大成效。加大金融、教育、就业、健康、科技等行业扶贫力度，完善社会保障体系。

全力推进污染防治。按照狠抓源头、精准治污、保护优先、从紧从严的要求，坚守阵地，巩固成果，全面完成中央环保督察“回头看”、自治区环境保护督察、自治区党委巡视反馈意见60个问题的整改。坚决打赢蓝天保卫战，扎实推进大气污染防治三年攻坚行动，推进落实关停小锅炉工作，盯住重点时段和重点区域，严格烟花爆竹和甘蔗叶禁燃管控，力争实现全年空气优良天数比例89%以上，PM2.5平均浓度控制在39微克/立方米以下。着力打好碧水保卫战，扎实推进水污染防治三年攻坚行动，推进重点行业清洁化改造、地下油罐改造、集中式饮用水水源地问题整改等工作，全面落实河长制、湖长制，确保地表水主要控制断面水质达标率100%，在用集中式饮用水水源地水质达标率100%。扎实推进净土保卫战，严格管控涉重金属行业企业污染，全面加强农业面源污染治理和土壤重金属污染防治及畜禽养殖粪污治理和资源化利用，推进重点区域环境综合整治和农村生活垃圾专项治理，进一步提升土壤环境质量。

（六）聚焦改革优化营商环境，蓄能激发高质量发展潜力

用好改革开放“关键一招”，以改革开放和营商环境大提升，激发经济高质量发展新活力。

深入推进供给侧结构性改革。落实“巩固、增强、提升、畅通”八字方针，持续强化“三去一降一补”，降低企业运行成本6亿元以上。贯彻落实“降成本政策落实年”，落实好自治区“41条”“28条”和我市“38条”“28条”降成本惠企政策，绝不能出现上面政策“很好”，下面办事“好狠”。继续深化电力体制改革，加快处置“僵尸企业”，推动企业兼并重组，推进产权、股权交易。畅通经济循环，提高金融服务实体经济能力，加强银企对接，开展“惠企贷”等活动，小微企业贷款余额增速达全区水平以上。深化市属国有企业改革，完善企业负责人薪酬、企业工资等机制体制，增强企业造血功能。统筹抓好农业农村、财税金融、医药卫生、养老服务、乡镇行政管理体制等领域改革，增强经济发展活力。

大力优化营商环境。贯彻落实“优化营商环境攻坚突破年”，深化“放管服”改革，完善提升“242530”改革，力争营商环境10项指标多次进入“红榜”，保持全区前列。大力推行“一事通办”，完成市政务中心升级改造，优化提升 “一站式”功能。推进“互联网+政务服务”改革，实施数字政务服务一体化建设，推动政务事项线上线下融合办理，实现24小时“不打烊”。建立互联网中介服务平台和实体中介超市，推动中介服务公开、规范、高效。深化商事登记制度改革，实现1个工作日完成企业开办；推进企业登记全程电子化，强化电子营业执照应用，实现企业开办“一网通办”。推行“一窗受理、集成服务”审批服务机制，各部门充分授权，不能让窗口的办事员“两头跑”、让办事的群众“跑两头”，真正做到“前台统一受理、后台分类审批、综合窗口出件”，力争95%以上的行政审批和公共服务事项“最多跑一次”。对我市来说，推动经济高质量发展，工业是龙头、是脊梁、是硬支撑，营商环境是形象、是保障、是软实力，我们将紧紧瞄准企业和群众的“最痛之点”“最盼之处”，抓住关键，直击短板，奋力推动营商环境优上加优、好上加好！

高质量开展精准招商。贯彻落实“产业大招商攻坚突破年”，主动融入自治区“南向、北联、东融、西合”开放发展新格局，抢抓“东融”发展战略机遇，创建深圳—来宾产业促进中心，密切跟踪粤港澳大湾区产业外溢发展动态，融入大湾区产业链、价值链、创新链，不断扩大来宾“朋友圈”。编制培植“工业树”、打造“产业林”招商计划，围绕“以资源换产业”和“一网两管”，对标我市传统产业“二次创业”和新兴产业壮大做强，通过以商招商、补链招商、驻点招商等方式，着力引进一批带动强龙头补链条的优势产业项目。推动龙头企业统筹经营连片开发，提升园区招商效率。在重点领域更注重招商选资，把招商引资选资与引才引智有机结合起来，着力推动和引领经济逐步迈向高质量发展。注重乡村招商“双回”工作，通过县干部带乡干部、乡干部带村干部，动员外出务工人员回乡投资办企业、回乡就近就业。认真抓好项目跟踪服务，确保引得进、落得地、建得好、见成效，力争全年引进亿元以上产业项目72个以上，到位资金增长15%以上。

坚持扩大有效投资。贯彻落实“重大项目建设攻坚突破年”， 完善中央预算内项目储备管理机制，加强用地和资金保障，力争获得建设用地指标8000亩、中央预算内补助资金5亿元，中央预算内投资项目年度进度达全区水平以上，力争进入“红榜”。抓好重大项目包装策划，超前谋划一批大项目、好项目，创新项目前期工作机制，挖掘包装更多使用者付费PPP项目。推动湘电风电制造、飞南铜资源环保再生利用等100个重大项目开工建设；推进桂中治旱、大藤峡工程等152个续建项目建设；实现象州理昂生物质发电、铭磊医药等52个重大项目竣工。力争重大项目投资增长12%，季度招商引资重点项目开竣工率及新签招商引资10亿元以上项目实施率达全区水平以上，力争进入“红榜”。

（七）聚焦城乡统筹，打造区域协调发展升级版

推动城乡统筹建设，提升区域发展水平，实现城镇化率达45.5%以上。

持续推进城镇化建设。坚持兴产业聚人气，实施大县城战略、产城融合战略和特色小镇战略，加强迁江镇经济发达镇行政管理体制改革，推进迁江铝特色小镇建设。深化以人为本的新型城镇化建设，促进农业转移人口市民化。持续推进城市管理执法体制改革，构建“大城管”格局；坚持城市精细化管理，加强卫生、牌匾广告、市场摊点等环境整治和“五乱”行为整治；推进数字化城市管理工作，完善市数字化城市管理中心系统平台9大标准核心系统，提升一体化处置能力和综合考评规范性。推进智慧物业管理，打造裕达社区等智慧物业示范社区。

持续优化人居环境。推进宜居城市建设，完善城市基础设施，加快城市污水处理厂建设步伐，对草鞋沟等城市黑臭水体进行综合整治；加快侨兴路、滨江北路西段等道路建设，打通维林大道延长线、翠屏东路等“断头路”。抓好桂中水城麒麟渠、水环境综合治理等重大项目建设。推进海绵城市建设，新增5%海绵城市建设面积。加大对市政道路、城市绿化等改造建设，实现建成区绿化覆盖率达34.5%。推进城市地下综合管廊建设，建设城镇燃气中压管道145公里，新建、改建市政公共厕所5座。继续实施“一企一策”，加快盘活处置市城区房地产停建项目；有序控制房地产开发，着力发展高品质房地产业，商品房销售面积增长10%以上。继续推进驻柳原柳地三大院的改造重建。

持续壮大县域经济实力。深入推进县域经济发展三年行动计划，着力做大产业、做强工业、做精招商、做实项目，推动县域经济全面发展。各县（市、区）、园区要按照以下目标推进：兴宾区要实现地区生产总值增长7.5%，规模以上工业增加值增长7%，招商引资到位资金增长15%，固定资产投资增长10%；象州县实现地区生产总值增长7%，规模以上工业增加值增长7%，招商引资到位资金增长15%，固定资产投资增长10%；武宣县实现地区生产总值增长7%，规模以上工业增加值增长7%，招商引资到位资金增长15%，固定资产投资增长10%；忻城县实现地区生产总值增长7%，规模以上工业增加值增长9%，招商引资到位资金增长15%，固定资产投资增长11%；金秀县实现地区生产总值增长7.5%，规模以上工业增加值增长7%，招商引资到位资金增长15%，固定资产投资增长18%；合山市实现地区生产总值增长7.5%，规模以上工业增加值增长10%，招商引资到位资金增长15%，固定资产投资增长10%；市工业园区实现规模以上工业增加值增长7%，招商引资到位资金增长15%，固定资产投资增长10%。

（八）聚焦共建共享，用心用情用力增进民生福祉

从人民群众的“烦恼事”“揪心事”抓起，把人民群众的小事当作我们的大事，一心一意做好民生工作。

推进教育共享。深入实施教育提升三年行动计划，有效整合、扩大教育资源，深入推进集团化、合作化、特色化、优质化办学，推进教育资源优质共享。实施来宾市普通高中教学质量提升行动计划，推进地区高中与象州县中、来宾高中与合山高中集团化办学。加快中职学校布局调整和专业结构优化，抓好星级学校建设培育工作。支持广西科技师范学院启动学校二期建设，推动高等教育内涵式发展。新（改、扩）建30所公办幼儿园，推进市九中、市十二中、富华小学等竣工验收，来宾一中迁建项目力争完成主体建设，加快普及十五年基本教育。全年实现学前三年毛入园率89%、义务教育巩固率95%、高中阶段毛入学率89.5%。

推进文体共享。推进60个村级公共服务中心项目建设，开展文艺文化惠民演出400场次，完成农村公益电影放映8700场以上。大力发展特色文化产业，储备文化产业项目10个，推进非物质文化遗产保护项目建设2个。着力抓好融媒体中心建设，建立“来宾云”融媒体生态系统，形成一盘棋，推动媒体整合。开展“扫黄打非”专项行动。以“体教融合”为重点，全力推进全民健身和全民健康深度融合试点城市建设，打造“全国全民健身示范城市”升级版，支持创意赛事产业发展，组织举办第十一届广西体育节来宾市分会场暨2019年来宾市全民健身运动会，完成学校、机关体育场馆对外开放5个以上。

推进医疗共享。全面推进健康素养促进行动计划，狠抓健康促进县和健康素养监测网络体系建设。深入推进分级诊疗体系建设，扩大县域医共体覆盖面，乡（镇）卫生院参与率达70%以上。选择1~2个县，由县级医院和辖区卫生院组建独立法人的县域医共体建设试点。扎实做好“互联网+医疗健康”工作，提升全市医疗卫生现代化水平。全力推进市人民医院创建“三甲”综合医院工作。扎实推进医养结合，实施医养结合项目3个，初步实现医养融合互动发展。全力做好自治区卫生城市复审工作。

加强社会保障。推进就业创业工作，实现城镇新增就业1万人，农村劳动力转移就业新增2.7万人次，建设扶贫车间50个以上。全力做好治欠保支工作，切实保障农民工合法权益。全面落实各项社保政策，完成社会保险参保的征缴任务。抓好救灾救济、优抚安置、社会福利和慈善等工作。完善住房保障体系，开工建设城市棚户区改造5995套，完成危房改造2210户。加快推进康养产业发展步伐，在市城区、县（市、区）各新增建设1个居家养老服务中心试点。实施10项市级为民办实事工程。

加强和创新社会治理。继续推进平安来宾建设。严厉打击涉黑恶违法犯罪活动，推动扫黑除恶专项斗争向纵深开展。加强法制建设，强化治安重点管控、公共安全防控和治安基础工作，突出抓好市域治理。继续打好禁毒人民战争。扎实做好信访和人民调解工作。严格落实安全生产责任制，深入开展安全风险管控和隐患排查治理，严防各类事故发生。加强消防救援队伍建设，着力提升社会应急联动和预警监测水平，提高突发事件应对能力，确保社会和谐稳定。

全面贯彻党的民族政策和宗教工作基本方针，促进民族团结、宗教和睦。做好军队退役人员安置和管理服务，巩固军政军民团结，再创全国双拥模范城。做好卫生健康、医疗保障工作，强化食品药品安全市场监管。扎实做好粮食和物资储备供应。深化统计改革，发挥统计信息、咨询、监督职能。充分发挥工会、共青团、妇联等人民团体桥梁纽带作用。

三、全面加强政府自身建设

心中有信仰，脚下有力量。信仰、信念、信心，任何时候都至关重要，我们将坚定政治信仰，坚持对人民负责、受人民监督、让人民满意，按照市委“提升全面从严治党水平”要求，持续推进政府自身建设，不断开创工作新局面。

铸忠诚，坚持旗帜鲜明讲政治。坚持用习近平新时代中国特色社会主义思想武装头脑，经常对表对标，及时校准偏差，增强“四个意识”、坚定“四个自信”、做到“两个维护”，自觉在思想上政治上行动上同以习近平同志为核心的党中央保持高度一致。坚决贯彻党中央对重大形势的科学判断和对重大工作的决策部署，全面贯彻落实自治区党委、政府和市委的决策部署，持续推进自治区党委巡视组巡视反馈意见的整改落实，切实履职尽责，凝聚和激发各方面积极性、主动性、创造性，精准发力推动工作。

重法治，坚持依法行政履好职。自觉接受市人大及其常委会的依法监督，积极支持市政协履行政治协商、民主监督、参政议政职能。认真办理人大代表建议和政协提案，做到事事有回音、件件有落实，进一步提高办理质量和满意度。加强与各民主党派、工商联、无党派人士和各人民团体的沟通协商。主动接受监察、审计、社会等方面监督。建立健全规范性文件合法性审查机制，坚持依法科学民主决策，严格规范公正文明执法，加强和改进行政复议、行政应诉工作，推动政府工作全面纳入法治轨道。

勇担当，坚持实干为本抓落实。坚持底线思维，提高防范化解重大风险能力。推动政府职能转变，按照市委的部署要求，加快推进市、县机构改革，确保3月底前完成改革任务。聚焦尚实干、勇作为、敢担当，坚持“严”字当头、“实”字托底，坚决反对庸政懒政、明哲保身、不作为乱作为。规范和改进督查考核工作，进一步精简文件、减少会议。大力开展“调查研究+”和“+调查研究”活动，以问题为导向，以目标为引领，带着工作、带着问题深入开展调查研究。建立工作倒逼机制，对重大部署、重要工作、重点工程，以“路线图”开路，以“清单表”推进，以“红黑榜”通报，推动工作落细落小，确保工作务实、过程扎实、结果真实。

葆廉洁，落实从严治党树形象。认真落实全面从严治党主体责任和意识形态工作责任制，严守政治纪律，严肃党内政治生活，严格执行民主集中制，带头贯彻中央八项规定及其实施细则精神，力戒形式主义、官僚主义，坚决整治隐形变异“四风”问题。强化权力制约监督，紧盯重大工程、重点领域、关键岗位，加强对财政资金使用、公共资源交易、国有资产管理、扶贫资金使用等的监督管理，坚定不移推进政府系统廉政建设和反腐败工作。

各位代表！幸福靠奋斗，来宾靠实干。让我们更加紧密地团结在以习近平同志为核心的党中央周围，在自治区党委、政府和市委的坚强领导下，坚定信心、一起奔跑，昼夜不舍、一起追梦，在建设壮美广西、共圆复兴梦想的新征程上，认真落实六“壮”六“美”要求，奋力推动来宾“壮”起来、“美”起来，以优异成绩庆祝新中国成立70周年！
